# Supplementary material for: C-Reactive Protein Is an Independent Predictor of 30-Day Bacterial Infection Post-Liver Transplantation
Source: Biomolecules. 2021 Aug 12;11(8):1195. doi: 10.3390/biom11081195 (PMC8391373; doi:10.3390/biom11081195)
Supplement: Supplementary file 1 [file biomolecules-11-01195-s001.zip › biomolecules-1300260-supplementary.pdf]

Supplementary Data

Table S1. Comparative analysis between patients with vs. without data of CRP.

Variable	Patients with Known CRP N = 940	Patients with Missing CRP N = 281	*p*-Value
CRP (mg/L)	8.47 (4.70–15.30)	(-)	
Age (years)	49 (41–56)	49 (43–56)	0.34
Female	168 (17.9%)	40 (14.2%)	0.155
MELD at transplantation	15.10 (10.01–25.62)	14.60 (9.12–24.40)	0.156
BMI (kg/m^2)	22.02 (19.83–24.22)	22.12 (19.84–24.39)	0.436
Sodium (mEq/L)	139 (136–141)	140 (136–142)	0.379
Serum albumin (g/L)	34.70 (31.58–38.60)	35.10 (31.80–38.30)	0.669
White blood cell(10^9/L)	4.80 (2.80–8.20)	4.90 (2.90–7.80)	0.87
NLR	3.86 (2.17–7.00)	3.77 (2.08–6.67)	0.548
SII	264 (130–577)	250 (135–560)	0.78
Donor age (years)	39 (29–49)	38 (30–48)	0.957
Blood loss (100 mL)	10 (8–18)	10 (8–17)	0.524
Liver tumor	390 (41.5%)	131 (46.6%)	0.127
**Organ failure number**			0.493
One or two	300 (31.9%)	80 (28.5%)	
Three or more	91 (9.7%)	26 (9.3%)	
Diabetes mellitus	152 (16.2%)	41 (14.6%)	0.524
Infection pre-transplant	126 (13.4%)	39 (13.9%)	0.838
Choledocho-jejunostomy	11 (1.17%)	5 (1.8%)	0.431
ABO incompatibility	139 (14.8%)	44 (15.7%)	0.72
CSI	209 (22.2%)	62 (22.1%)	0.952

Abbreviations, CSI, clinically significant bacterial infection; CRP, C-reactive protein; NLR, neutrophil-lymphocyte ratio; SII, systemic immune-inflammation index.

Table S2. Systemic inflammatory response markers in patients separated by infection pre-transplant.

Variable	Infection Pre-Transplant (No) N = 814	Infection Pre-Transplant (Yes) N = 126
WBC (10^9/L)	4.70 (2.70–7.68)	5.95 (3.52–11.15) *
	HR 1.06 (95%CI, 1.05–1.08) $p < 0.001$	HR 1.06 (95%CI, 1.01–1.11) $p = 0.013$
NLR	3.64 (2.05–6.58)	5.47 (3.34–10.97) *
	HR 1.0745 (95%CI,1.05–1.10) $p < 0.001$	HR 1.02 (95%CI, 0.99–1.04) $p = 0.138$
SII	247 (127–547)	394 (186–738) *
	HR 1.0005 (95%CI, 1.0003–1.0007) $p < 0.001$	HR 1.0001 (95%CI, 0.9997–1.0005) $p = 0.744$
CRP(mg/L)	8.21 (4.44–13.85)	11.45 (6.79–24.24) *
	HR 1.02 (95%CI, 1.01, 1.03) $p < 0.001$	HR 1.02 (95%CI, 1.01, 1.03) $p = 0.003$

Abbreviations, CRP, C-reactive protein; NLR, neutrophil-lymphocyte ratio; WBC, white blood cell SII, systemic immune-inflammation index; HR, hazard ratio; CI, Confidence interval. * *p* < 0.01, compared with patients without infection pre-transplant.

Table S3. Systemic inflammatory response markers in patients separated by organ failure number.

Variable	Organ Failure Number (None) N = 549	Organ Failure Number (One or two) N = 300	Organ Failure Number (Three or more) N = 91
WBC (10^9/L)	3.50 (2.30–5.10)	7.20 (4.80–10.93) *	10.60 (8.20–13.20) *
	HR 1.07 (95%CI, 1.01–1.13) *p* = 0.013	HR 1.03 (95%CI, 1.01–1.06) *p* = 0.019	HR 1.005 (95%CI,0.95–1.06) *p* = 0.854
NLR	2.83 (1.71–4.43)	5.61 (3.48–8.47) *	8.82 (5.75–12.62) *
	HR 1.04 (95%CI, 0.9997–1.089) *p* = 0.052	HR 1.01 (95%CI, 0.98- 1.04) *p* = 0.447	HR 1.02 (95%CI, 0.995–1.049) *p* = 0.112
SII	176 (106–349.19)	429 (228–817) *	590 (306–913) *
	HR 1.0006 (95%CI, 1.0003–1.0010) *p* < 0.001	HR 1.0001 (95%CI, 0.9998–1.0004) *p* = 0.498	HR 1.0000 (95%CI, 0.9995–1.0005) *p* = 0.930
CRP(mg/L)	6.80 (3.80–13.00)	10.47 (6.97–18.23) *	8.50 (5.70–16.10) *
	HR 1.02 (95%CI, 1.015–1.03) *p* < 0.001	HR 1.02 (95%CI, 1.01–1.03) *p* = 0.001	HR 1.02 (95%CI, 1.003, 1.027) *p* = 0.014

Abbreviations, CRP, C-reactive protein; NLR, neutrophil-lymphocyte ratio; WBC, white blood cell SII, systemic immune-inflammation index; HR, hazard ratio; CI, Confidence interval; * *p* < 0.05 compared with patients without organ failure.

Table S4. Systemic inflammatory response markers in patients separated by MELD score quartile.

Variable	MELD Quartile 1 (0.22–9.73) N = 222	MELD Quartile 2 (9.76–14.96) N = 243	MELD Quartile 3 (15.00–25.26) N = 235	MELD Quartile 4 (25.31–40.00) N = 240
WBC (10^9/L)	4.25 (3.00–5.60)	3.00 (2.10–4.80) **	4.80 (2.65–8.45) *	9.00 (6.00–12.10) **
	HR 1.16 (95%CI, 1.03–1.30) *p* = 0.017	HR 1.02 (95%CI, 0.87- 1.20) *p* = 0.777	HR 1.06 (95%CI, 1.02- 1.10) *p* = 0.005	HR 1.03 (95%CI, 1.00- 1.05) *p* = 0.024
NLR	2.60 (1.64–4.10)	3.17 (2.00–4.71) *	4.33 (2.35–7.23) **	7.10 (4.00–10.91) **
	HR 1.11 (95%CI, 1.00–1.22) *p* = 0.043	HR 1.01 (95%CI, 0.93- 1.09) *p* = 0.815	HR 1.03 (95%CI, 1.00- 1.07) *p* = 0.051	HR 1.03 (95%CI, 1.01- 1.05) *p* = 0.009
SII	230 (137.88–405.98)	162.00 (89.17–358.78) **	279.50 (118.52–604.63)	442.66 (249.46–854.83) **
	HR 1.0006 (95%CI, 1.0001–1.0010) *p* = 0.018	HR 1.0004 (95%CI, 0.9999–1.0009) *p* = 0.108	HR 1.0004 (95%CI, 1.0001–1.0006) *p* = 0.005	HR 1.0001 (95%CI, 0.9998–1.0004) *p* = 0.598
CRP(mg/L)	8.21 (3.50–14.53)	6.60 (3.88–13.34)	8.80 (5.16–14.77)	9.89 (6.25–16.97) **
	HR 1.02 (95%CI, 1.01–1.03) *p* =	HR 1.02 (95%CI, 1.00–1.04) *p* =	HR 1.03 (95%CI, 1.02, 1.05) *p* <	HR 1.02 (95%CI, 1.02, 1.03) *p* <

	0.003	0.048	0.001	0.001

Abbreviations, CRP, C-reactive protein; NLR, neutrophil-lymphocyte ratio; WBC, white blood cell SII, systemic immune-inflammation index; HR, hazard ratio; CI, Confidence interval; * $p < 0.05$; ** $p < 0.01$ compared with patients at MELD quartile 1.

Table S5. Systemic inflammatory response markers in patients separated by liver tumor.

Variable	Liver Tumor (No) N = 550	Liver Tumor (Yes) N = 390
WBC (10^9/L)	5.80 (3.10–9.97)	4.00 (2.70–5.60) *
	HR 1.05 (95%CI, 1.04–1.07) $p < 0.001$	HR 1.06 (95%CI, 1.01–1.12) $p = 0.029$
NLR	4.71 (2.52–8.17)	3.00 (1.88–4.96) *
	HR 1.04 (95%CI, 1.03–1.06) $p < 0.001$	HR 1.04 (95%CI, 0.99, 1.09) $p = 0.102$
SII	324 (128–625)	215 (137–439) *
	HR 1.0004 (95%CI, 1.0002–1.0005) $p < 0.001$	HR 1.0005 (95%CI, 1.0002–1.0008) $p < 0.001$
CRP(mg/L)	8.80 (5.20–14.20)	7.98 (3.92–16.72)
	HR 1.03 (95%CI,1.02, 1.04) $p < 0.001$	HR 1.02 (95%CI,1.01, 1.03) $p < 0.001$

Abbreviations, CRP, C-reactive protein; NLR, neutrophil-lymphocyte ratio; WBC, white blood cell SII, systemic immune-inflammation index; HR, hazard ratio; CI, Confidence interval. * $p < 0.01$, compared with patients without liver tumor.

Table S6. Exclusion of diagnosis CSI within 1, 2, and 5 day(s) after inclusion in the cohort.

Variable	Exclusion CSI Diagnoses < 1 days HR (95% CI) N = 920	Exclusion CSI Diagnoses < 2 days HR (95% CI) N = 895	Exclusion CSI Diagnoses < 5 days HR (95% CI) N = 831
NLR	1.052 (1.037–1.067) $p < 0.001$	1.0506 (1.032–1.070) $p < 0.001$	1.044 (1.019–1.069) $p < 0.001$
SII	1.0004 (1.0003–1.0006) $p < 0.001$	1.0004 (1.0003–1.0003) $p < 0.001$	1.0004 (1.0001–1.0006) $p = 0.005$
WBC(10^9/L)	1.064 (1.049–1.079) $p < 0.001$	1.068 (1.052–1.084) $p < 0.001$	1.095 (1.063–1.127) $p < 0.001$
CRP(mg/L)	1.021 (1.015–1.026) $p < 0.001$	1.021 (1.015–1.027) $p < 0.001$	1.023 (1.015–1.030) $p < 0.001$

Abbreviations, CSI, clinically significant bacterial infection; CRP, C-reactive protein; NLR, neutrophil-lymphocyte ratio; SII, systemic immune-inflammation index; WBC, White blood cell; HR, hazard ratio; CI, Confidence interval.

Table S7. Multivariate analysis of risk factors for 30-day CSI compared with the result of competing risk model.

Variable	Competing Risk Model Analysis	
	**HR (95% CI)**	***p*-Value**
NLR	1.03 (0.99, 1.06)	0.111
Choledocho-jejunostomy	7.56 (3.02, 18.89)	<0.001
MELD at transplantation	1.03 (0.99, 1.06)	0.154
Organ failure number (None)	Reference	
One or two	1.67 (0.85, 3.28)	0.136
Three or more	4.22 (1.73, 10.32)	0.002
CRP (mg/L)	1.02 (1.01, 1.03)	<0.001

Abbreviations, CSI, clinically significant bacterial infection; CRP, C-reactive protein.

Table S8. Performances of systemic inflammatory response markers and MELD score in the diagnosis of CSI after transplantation.

Variable	AUC(95%CI)	Best Threshold	Specificity	Sensitivity	Postive-pv	Negative-pv	*p*-Value *
CRP (mg/L)	0.742(0.693, 0.791)	9.48	0.631	0.800	0.388	0.915	-
WBC (10^9/L)	0.683(0.625, 0.740)	7.55	0.796	0.530	0.433	0.853	0.104 *
NLR	0.679(0.619, 0.738)	6.94	0.817	0.504	0.446	0.849	0.090 *
SII	0.672(0.616, 0.727)	389	0.692	0.626	0.373	0.864	0.038 *
MELD at transplantation	0.684(0.622, 0.745)	21.3	0.713	0.635	0.393	0.870	0.158 *

Abbreviations, CSI, clinically significant bacterial infection; CRP, C-reactive protein; NLR, neutrophil-lymphocyte ratio; SII, systemic immune-inflammation index; WBC, White blood cell; CI, Confidence interval; Positive PV: positive predictive value; Negative PV: negative predictive value. * compared with the performance of CRP.

Table S9. Comparative Analysis between patients with vs. without LPS Data.

Variable	Patients with Known LPS N = 940	Patients with Missing LPS N = 80	*p*-Value
Plasma LPS (EU/mL)	(-)	0.34 (0.06–0.92)	(-)
CRP (mg/L)	8.54 (4.60–15.60)	8.05 (5.47–11.99)	0.65
Age (years)	50 (42–57)	45 (38–54)	0.005
Female	157 (18.26%)	11 (13.75%)	0.314
MELD at transplantation	14.08 (9.59–23.32)	27.79 (25.17–31.33)	<0.001
BMI (kg/m2)	21.97 (19.83–24.22)	22.48 (20.04–24.57)	0.568
Sodium (mEq/L)	139 (136–141)	138 (136–141)	0.636
Serum albumin (g/L)	35.00 (31.70–39.00)	32.95 (30.37–35.23)	<0.001
White blood cell(10^9/L)	4.50 (2.70–7.20)	9.50 (7.20–12.40)	<0.001
NLR	3.57 (2.07–6.57)	7.22 (5.08–10.01)	<0.001
SII	241 (125–507)	620 (352–976)	<0.001
Donor age (years)	37.30 (28.00–48.00)	46.95 (38.72–51.85)	<0.001
Blood loss (100 mL)	10 (8–20)	8 (6–11)	0.001
Liver tumor	389 (45.23%)	1 (1.25%)	<0.001
**Organ failure number**			<0.001
One or two	246 (28.60%)	54 (67.50%)	
Three or more	65 (7.56%)	26 (32.50%)	

Diabetes mellitus	141 (16.40%)	11 (13.75%)	0.539
Infection pre-transplant	114 (13.26%)	12 (15.00%)	0.661
Choledocho-jejunostomy	11 (1.28%)	0 (0.00%)	0.309
ABO incompatibility	112 (13.02%)	27 (33.75%)	<0.001
CSI	183 (21.28%)	26 (32.50%)	0.021

Abbreviations, LPS, lipopolysaccharide; CSI, clinically significant bacterial infection; CRP, C-reactive protein; NLR, neutrophil-lymphocyte ratio; SII, systemic immune-inflammation index.

Table S10. Association between LPS level pre-transplant and 30-day CSI after transplantation.

Variable	No CSI N = 54	CSI N = 26	HR (95% CI)	*p*-Value
LPS (EU/mL)	0.23 (0.04–0.69)	0.72 (0.15–1.44) *	1.30 (1.01, 1.68)	0.041

Abbreviations, LPS, lipopolysaccharide; CSI, clinically significant bacterial infection; HR, hazard ratio; CI, Confidence interval; *$p < 0.05$ compared with patients of no CSI.

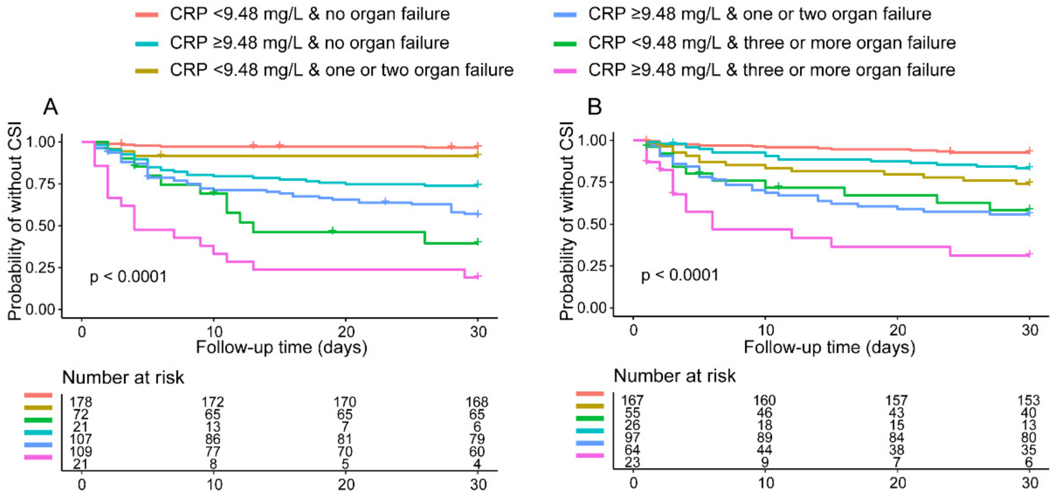

Figure S1. Kaplan-Meier curves illustrating the differences in 30-day CSI in groups stratified by CRP cutoff value of 9.48 mg/L and organ failure number. (**A**) Exploratory group; (**B**) validation group.

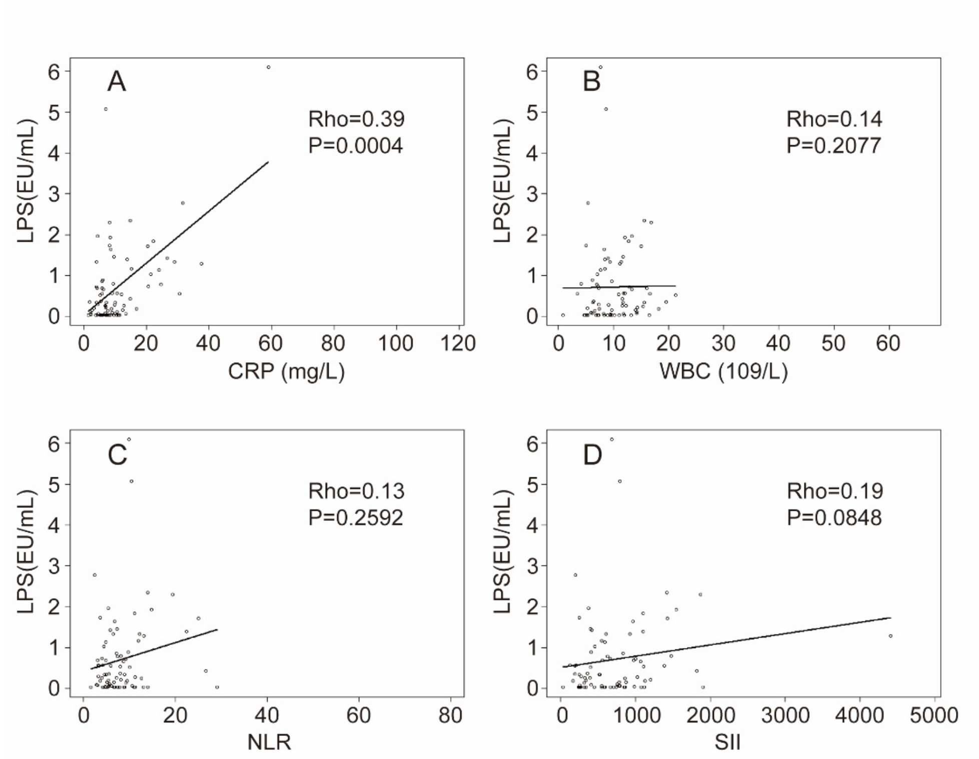

Figure S2. Correlation of LPS (lipopolysaccharide) with selected systemic inflammatory response markers. Abbreviations, LPS, lipopolysaccharide; CRP, C-reactive protein; WBC, White blood cell; NLR, neutrophil-lymphocyte ratio; SII, systemic immune-inflammation index.
